# ROS enhance angiogenic properties via regulation of NRF2 in tumor endothelial cells

**DOI:** 10.18632/oncotarget.17567

**Published:** 2017-05-02

**Authors:** Takayuki Hojo, Nako Maishi, Alam Mohammad Towfik, Kosuke Akiyama, Noritaka Ohga, Masanobu Shindoh, Yasuhiro Hida, Kazuyuki Minowa, Toshiaki Fujisawa, Kyoko Hida

**Affiliations:** ^1^ Vascular Biology, Frontier Research Unit, Institute for Genetic Medicine, Hokkaido University, Sapporo 060-0815, Japan; ^2^ Department of Dental Anesthesiology, Hokkaido University Graduate School of Dental Medicine, Sapporo 060-8586, Japan; ^3^ Department of Dental Radiology, Hokkaido University Graduate School of Dental Medicine, Sapporo 060-8586, Japan; ^4^ Department of Oral Diagnosis and Medicine, Hokkaido University Graduate School of Dental Medicine, Sapporo 060-8586, Japan; ^5^ Department of Oral Pathology and Biology, Hokkaido University Graduate School of Dental Medicine, Sapporo 060-8586, Japan; ^6^ Department of Cardiovascular and Thoracic Surgery, Hokkaido University Graduate School of Medicine, Sapporo 060-8638, Japan

**Keywords:** tumor angiogenesis, tumor endothelial cells, reactive oxygen species, biglycan, nuclear factor erythroid 2-related factor 2

## Abstract

Reactive oxygen species (ROS) are unstable molecules that activate oxidative stress. Because of the insufficient blood flow in tumors, the tumor microenvironment is often exposed to hypoxic condition and nutrient deprivation, which induces ROS accumulation. We isolated tumor endothelial cells (TECs) and found that they have various abnormalities, although the underlying mechanisms are not fully understood. Here we showed that ROS were accumulated in tumor blood vessels and ROS enhanced TEC migration with upregulation of several angiogenesis related gene expressions. It was also demonstrated that these genes were upregulated by regulation of Nuclear factor erythroid 2-related factor 2 (NRF2). Among these genes, we focused on *Biglycan*, a small leucine-rich proteoglycan. Inhibition of Toll-like receptors 2 and 4, known BIGLYCAN (BGN) receptors, cancelled the TEC motility stimulated by ROS. ROS inhibited NRF2 expression in TECs but not in NECs, and NRF2 inhibited phosphorylation of SMAD2/3, which activates transcription of BGN. These results indicated that ROS-induced BGN caused the pro-angiogenic phenotype in TECs via NRF2 dysregulation.

## INTRODUCTION

Tumor angiogenesis, the formation of new blood vessels in tumor tissues, supplies oxygen and nutrients to tumors and is required for tumor progression [1]. Tumor cells induce angiogenesis by secreting several growth factors and cytokines [2]. However, tumor tissues contain hypoxic and nutrient-starved microenvironment [3]. Because of the rapid growth of tumors, blood vessels in tumor tissues show immature morphology with lack of pericyte coverage and loss of endothelial–endothelial cell contact, which causes high vessel permeability and increases the tumor interstitial fluid pressure. These phenomena lead to chaotic blood vessel patterns and disorganized circulation.

When tissues are exposed to hypoxia and undernutrition, reactive oxygen species (ROS) are accumulated [4, 5]. ROS production is one of the causes of oxidative stress, and excessive ROS levels lead to cell death [6]. However, in normal cells, a low level of ROS is required for the signal transduction involved in cell migration and proliferation [7]. In cancer cells, on the other hand, a high concentration of ROS maintains the high cell proliferation rate with accelerated metabolism. Previous studies have shown that ROS accumulation by commonly used radio- and chemotherapeutic drugs influences their malignancy [6, 8]. How to regulate ROS level in cancer therapies has not been determined.

We previously isolated endothelial cells from tumor tissues (tumor endothelial cells, TECs) and found that TECs differ from normal endothelial cells (NECs) in several aspects. For example, TECs proliferate and migrate faster than NECs and show upregulation of several genes such as *Biglycan* [9–16]. TECs are also resistant to several anti-cancer drugs [17–19] and exhibit aneuploidy [20, 21]. We previously found that endothelial cells could acquire aneuploidy in hypoxic conditions via ROS accumulation [22]. A previous report showed that TECs are resistant to ROS with activation of ataxia telangiectasia mutated (ATM) kinase by ROS stimulation [23]. However, the mechanism underlying the response to oxidative stress in TECs is still not completely known.

BIGLYCAN (BGN), a small leucine-rich proteoglycan, is a component of the extracellular matrix and is secreted from various cells in inflammatory tissues [24–26]. BGN is one of the damage-associated molecular patterns (DAMPs), which are endogenous molecules that are released from the intracellular or extracellular space following tissue stress. DAMPs trigger the immune response via Toll-like receptor (TLR) 2, TLR4 and other factors [27, 28]. We previously found that *Biglycan* is highly expressed in TECs compared with NECs [12]. BGN functions in the migration of TECs in an autocrine manner through TLR2 and TLR4 [12]. In addition, BGN secreted from TECs attracts tumor cells to metastasize in a paracrine manner via activation of nuclear factor-κB (NF-κB) and extracellular signal-regulated kinase 1/2 (ERK1/2) [29]. Recent studies showed that BGN is elevated in tumor tissues such as colon cancer [30], and increased BGN expression is correlated with poor prognosis [31]. BGN has gathered attention because of its potential as a target for anticancer therapies.

Nuclear factor erythroid 2-related factor 2 (NRF2) is kept inactive under basal conditions by Kelch-like ECH-associated protein 1, which binds to NRF2 and promotes its degradation by the ubiquitin proteasome pathway [32–35]. NRF2 protects cells from oxidative and inflammatory stress by upregulating the expression of cytoprotective genes [32–35]. A recent report showed that NRF2 negatively regulates the phosphorylation of SMAD2/3 and that loss of NRF2 leads to SMAD2/3 phosphorylation [36, 37]. NRF2 in endothelial cells was reported to be involved in angiogenesis [38]. However, the function of NRF2 in endothelial cells in tumors is unknown.

In this study, we examined the role of ROS in TECs as well as the potential involvement of NRF2 and BGN.

## RESULTS

### ROS accumulation in tumor blood vessels

**Figure 1:**
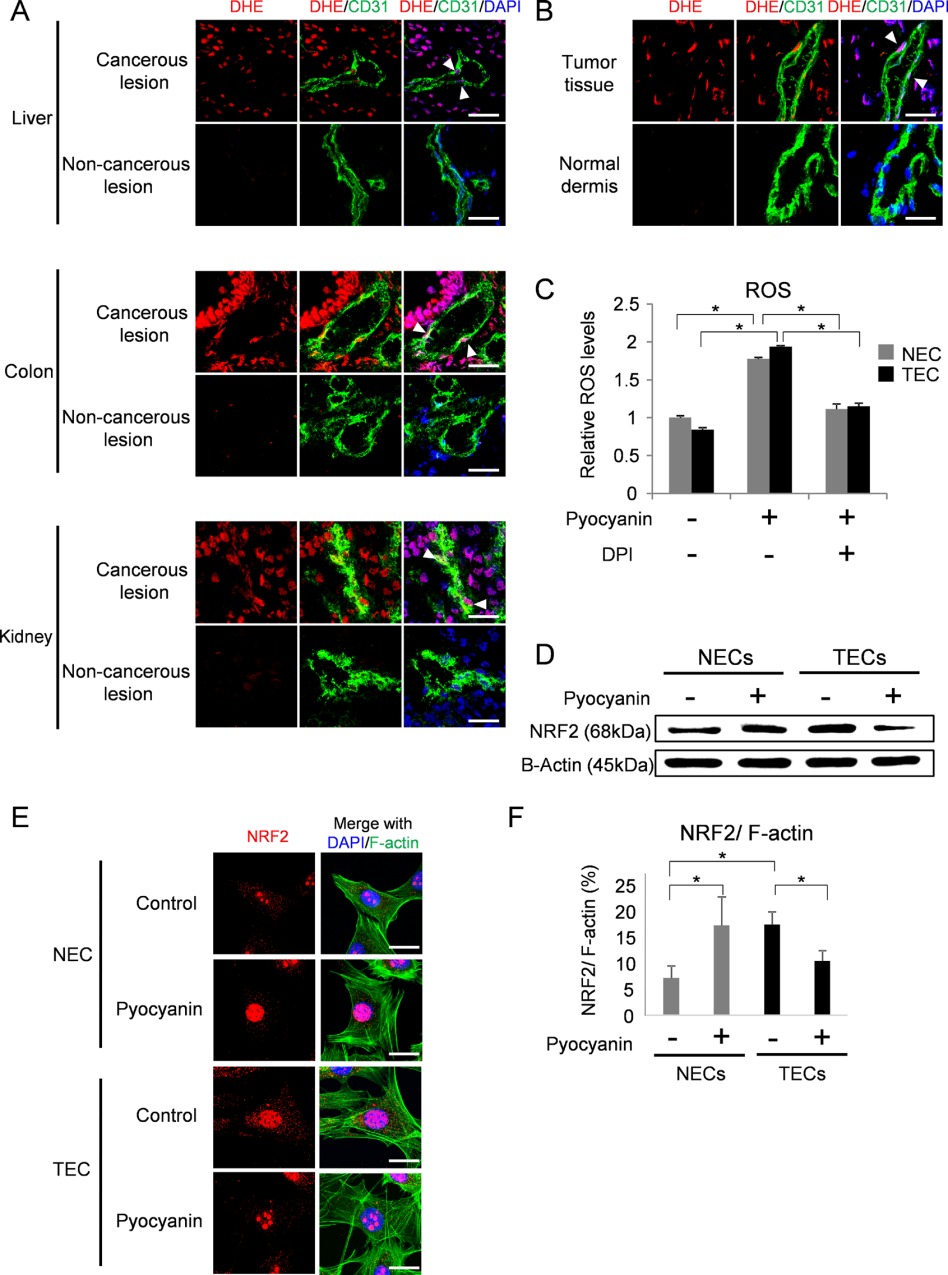
NRF2 response to ROS accumulation in endothelial cells (**A**) ROS were visualized with DHE staining (red) in cancerous lesions and non-cancerous lesions of human hepatocellular carcinoma (liver), colorectal cancer (colon) and renal cell carcinoma (kidney). Merged images stained with anti-CD31 antibody (green) and DAPI (blue) are shown in the middle and right panels. Arrowhead indicates co-localization of DHE and CD31. Scale bar = 30 μm. (**B**) ROS were visualized with DHE staining (red) in A375-SM xenografts and normal dermis of nude mice. Merged images with CD31 (green) and nuclei stained with DAPI (blue) are shown. Arrowhead indicates co-localization of DHE and CD31. Scale bar = 20 μm. (**C**) Relative ROS levels in NECs (gray columns) and TECs (black columns). *P < 0.01, one-way ANOVA. Data are represented as mean ± SD, *n* = 6. (**D**) NRF2 protein levels in NECs and TECs with or without pyocyanin treatment (25 μM) were analyzed by western blotting. B-Actin served as loading control. (**E**) Immunocytochemistry for NRF2 (red) with DAPI (blue) and F-actin (green) staining in NECs and TECs with or without pyocyanin treatment (25 μM). Scale bar = 20 μm. (**F**) Quantitative expression of NRF2 in NECs and TECs with or without pyocyanin treatment (25 μM). F-actin was used for normalization. *P < 0.05, one-way ANOVA. Data are represented as mean ± SD, *n* = 50 cells.

ROS are reported to be aberrantly accumulated in tumor tissues [6, 39]. We visualized ROS in human tumor tissues by staining with dihydroethidium (DHE) (Figure 1A). ROS signals were accumulated in tumor blood vessels (Figure 1A, arrow heads). In contrast, DHE was hardly stained in blood vessels of the normal counterparts. Similarly, DHE signals were detected in blood vessels of human tumor xenografts grown in nude mice but not in those of normal dermis (Figure 1B). These data suggest that tumor tissues are exposed to more oxidative stress than normal tissues, resulting in increased ROS production in tumor tissues, including tumor blood vessels.

To elucidate the effect of oxidative stress on endothelial cells, we used cultured NECs and TECs isolated from normal dermis and tumor xenografts, respectively, as previously described [9, 12, 14–20, 40]. ROS levels were almost the same in NECs and TECs in culture (Figure 1C, Supplementary Figure 1A and 1B). Consistent with previous reports [6, 39], both NECs and TECs treated with the ROS inducer pyocyanin and/or ROS scavenger diphenyleneiodonium (DPI) showed similar changes in ROS levels (Figure 1C). Similarly, ROS levels were almost the same between NECs and TECs in hypoxia (Supplementary Figure 1A) and in serum starved conditions and (Supplementary Figure 1B). These results suggest that ROS levels showed similar responses in cultured endothelial cells from tumor tissues compared with those non-tumor tissues.

### Response of NRF2 to ROS in NECs and TECs

NRF2 mediates cellular antioxidant responses [32–35]. We next analyzed NRF2 levels in NECs and TECs by western blotting. TECs showed higher levels of NRF2 expression compared with NECs under untreated conditions (Figure 1D). NRF2 is a transcription factor. We next analyzed the expression of *Ho-1*, which is regulated by NRF2. *Ho-1* was higher in TECs than in NECs as expected (Supplementary Figure 1C). Consistent with previous reports [33], NRF2 levels were increased in NECs treated with the ROS-inducer pyocyanin (Figure 1D). However, in TECs treated with pyocyanin, NRF2 levels were reduced. Similarly, immunocytochemistry showed that pyocyanin induced NRF2 expression in NECs, while pycocyanin reduced NRF2 in TECs (Figure 1E and 1F). As described above, TECs show abnormal phenotypes, but the mechanism underlying these abnormalities has not been determined. We thus further focused on the NRF2 variation in TECs in response to ROS accumulation to elucidate these abnormalities.

### SMAD2/3 are regulated by NRF2 in TECs

We previously reported that TECs exhibit more pro-angiogenic properties compared with NECs with upregulation of several genes, including *Biglycan* [10–16, 40]. Since the expression of BGN is regulated by SMAD2/3 [41], and SMAD2/3 are regulated by NRF2 [36, 37], it is conceivable that ROS accumulation affects BGN expression through NRF and SMAD2/3 in TECs.

**Figure 2:**
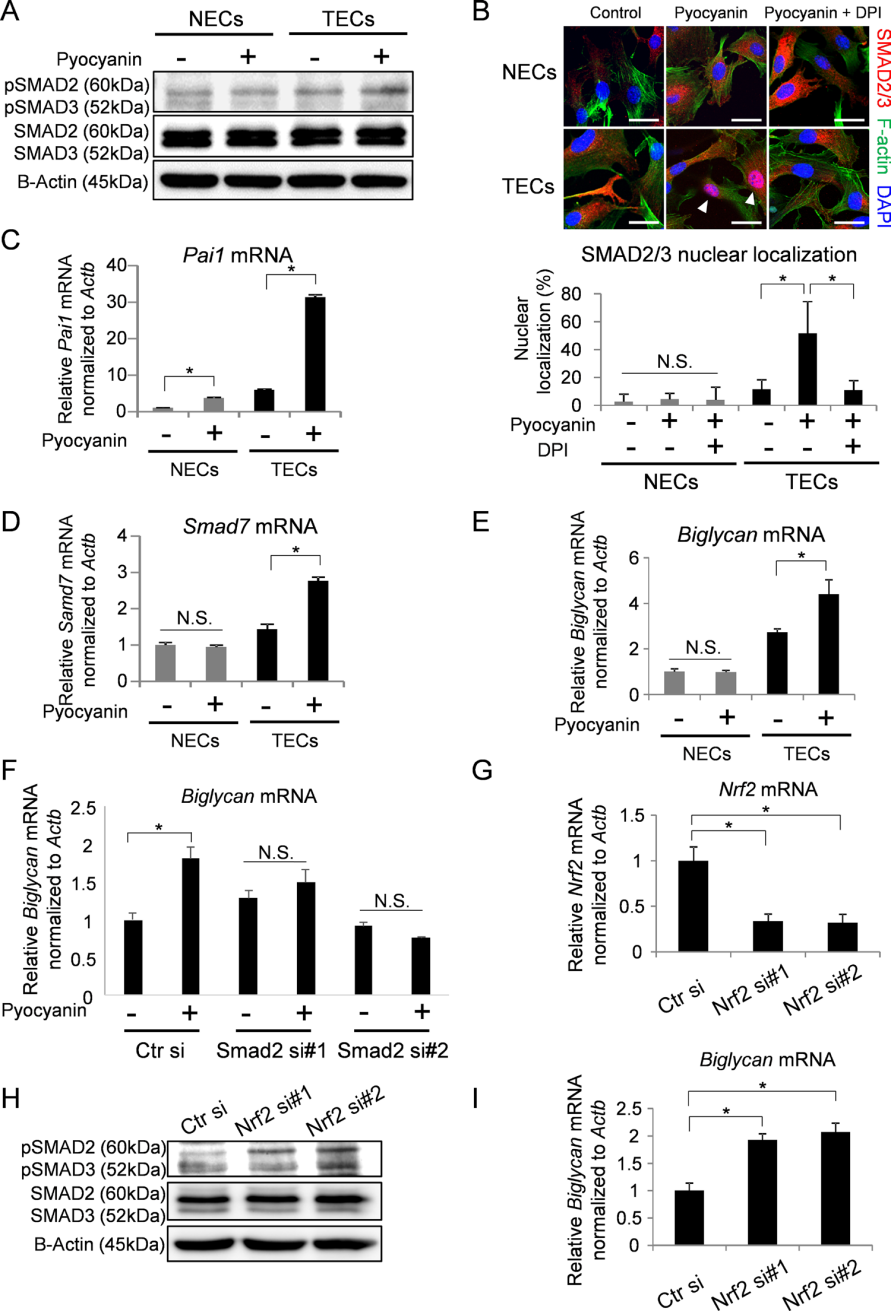
SMAD2/3 were regulated by NRF2 in TECs followed by upregulation of BGN expression (**A**) Phosphorylated SMAD2 and 3 (pSMAD2/3) and total SMAD2 and 3 (SMAD2/3) levels were analyzed in cells stimulated with pyocyanin (25 μM) by western blotting. B-Actin served as loading controls. (**B**) Immunocytochemistry for SMAD2/3 (red) with DAPI (blue) and F-actin (green) in cells treated with pyocyanin (25 μM) and DPI (1 μM). Scale bar = 20 μm. Arrowhead indicates localization of SMAD2/3 in nuclei (DAPI stained-area). Quantification of nuclear localization of SMAD2/3 is shown in the graph. *P < 0.01, one-way ANOVA. N.S., not significant. Data are represented as mean ± SD, *n* = 50 cells. (**C**, **D**) *Pai1* mRNA (C) and *Smad7* mRNA (D) expressions were evaluated by real-time PCR. *P < 0.01, one-way ANOVA. Data are mean ± SD, *n* = 4 real-time RT-PCR runs. (**E**) *Biglycan* mRNA in NECs and TECs with or without pyocyanin treatment (25 μM) were analyzed by real-time PCR. *P < 0.01, N.S., not significant. One-way ANOVA. Data are mean ± SD, *n* = 4 real-time RT-PCR runs. (**F**) *Biglycan* mRNA in *Smad2* knock-down TECs with or without pyocyanin treatment (25 μM) were analyzed by real-time PCR. *P < 0.01, N.S., not significant. One-way ANOVA. Data are mean ± SD, *n* = 4 real-time RT-PCR runs. (**G**) Real-time PCR confirms silenced *Nrf2* mRNA levels. *P < 0.01, one-way ANOVA. Data are mean ± SD, *n* = 4 real-time RT-PCR runs. (**H**) pSMAD2/3 and SMAD2/3 levels in NRF2 knock-down TECs were analyzed by western blotting. B-Actin served as loading control. (**I**) *Biglycan* mRNA in NRF2 silenced TECs were analyzed by real-time PCR. *P < 0.01, one-way ANOVA. Data are mean ± SD, *n* = 4 real-time RT-PCR runs.

Induction of ROS by pyocyanin treatment showed that SMAD2 were activated in TECs but not NECs (Figure 2A). It is known that activated SMAD2/3 are localized in nuclei [42]. Staining of pyocyanin-treated TECs revealed SMAD2/3 in nuclei, and this localization was abrogated by DPI treatment (Figure 2B). To further confirm the activation of the SMAD pathway in TECs, the expressions of *Pai-1* and *Smad7*, which are downstream members of the SMAD pathway, were analyzed by real-time PCR. As we expected, both *Pai1* and *Smad7* mRNA levels in TECs were elevated by pyocyanin treatment (Figure 2D). However, pyocynin treatment also increased the expression of *Pai1* in NECs (Figure 2C). *Pai1* is induced by ERK activation [43] and ERK is activated by ROS generation [7, 43]. We thus next analyzed the activation of ERK with ROS induction. Our results showed that ERK was phosphorylated in both NECs and TECs after pyocyanin treatment (Supplementary Figure 2A). Furthermore, increase in the *Pai1* mRNA expression level was canceled by ERK inhibition (Supplementary Figure 2B). These data suggest that *Pai1* mRNA expression was induced by both Smad2 and ERK activation with ROS generation. Furthermore, the expression of *Biglycan* mRNA was upregulated in TECs by pyocyanin treatment, but not in NECs (Figure 2E). When *Smad2* was knocked down by siRNA in TECs (Supplementary Figure 2C), *Biglycan* upregulation by ROS accumulation was blocked (Figure 2F). These data suggest that *Biglycan* upregulation by ROS accumulation may be due to the activation of SMAD2/3.

We also examined the contribution of NRF2 to SMAD activation in TECs. When *Nrf2* was knocked down by siRNA (Figure 2G), SMAD2/3 were phosphorylated (Figure 2H) and *Biglycan* mRNA was upregulated (Figure 2I). These data suggest that ROS accumulation reduces NRF2 levels in TECs, causing activation of SMAD2/3 and subsequent upregulation of *Biglycan* mRNA expression.

### BGN induced by ROS activates cell motility in TECs

**Figure 3:**
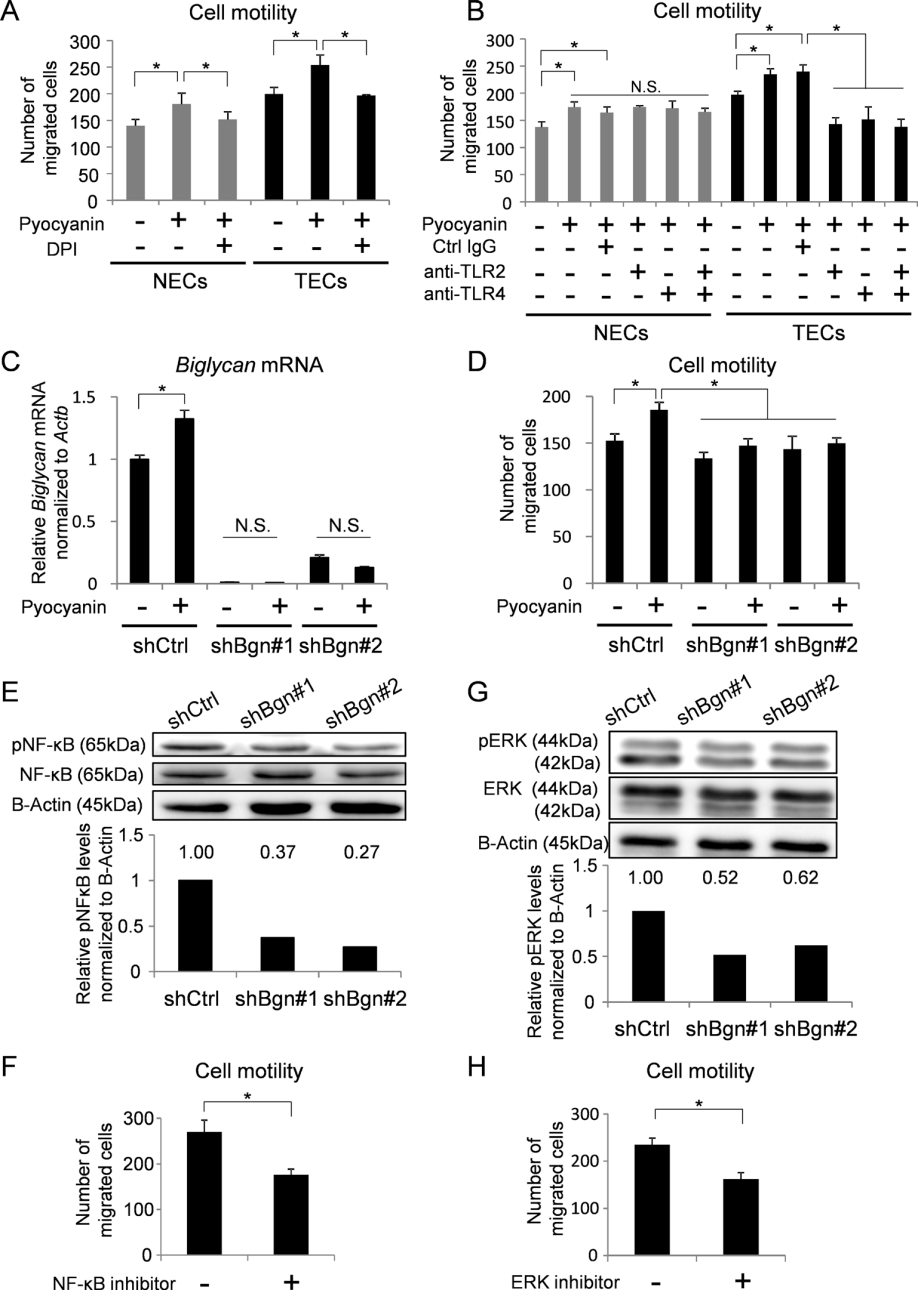
BGN induced by ROS activates cell motility in TECs (**A**) Cell motilities of NECs and TECs treated with pyocyanin (25 μM) and DPI (1 μM) were analyzed using a Boyden chamber. *P < 0.01, one-way ANOVA. Data are represented as mean ± SD, *n* = 4 fields. (**B**) Cell motilities of NECs and TECs treated with pyocyanin (25 μM) were analyzed by Boyden chamber migration assay in the presence of 20 μM of control (Ctrl) IgG or anti-TLR2 antibody or anti-TLR4 antibody. *P < 0.01, one-way ANOVA. N.S., not significant. Data are represented as mean ± SD, *n* = 4 fields. (**C**) *Biglycan* mRNA in indicated TECs were analyzed by real-time PCR. *P < 0.01, two-sided Student's *t*-test. Data are mean ± SD, *n* = 4 real-time RT-PCR runs. (**D**) Cell motilities of indicated TECs treated with pyocyanin (25 μM) were analyzed by Boyden chamber migration assay. *P < 0.01, one-way ANOVA. Data are represented as mean ± SD, *n* = 4 fields. (**E**) Phosphorylated NF-κB (pNF-κB) and total NF-κB (NF-κB) in BGN knockdown TECs were analyzed by western blotting. B-Actin served as internal control. Levels of pNF-κB were normalized to B-actin using densitometry. (**F**) TEC motility in the presence of 10 μM of the NF-κB inhibitor, BAY11-7082, was evaluated using a Boyden chamber. *P < 0.01, one-way ANOVA. Data are represented as mean ± SD, *n* = 4 fields. (**G**) Phosphorylated ERK (pERK) and total ERK (ERK) in BGN knockdown TECs were analyzed by western blotting. B-Actin served as loading control. Levels of pERK were normalized to B-actin using densitometry. (**H**) TEC motility in the presence of 10 μM of the MEK inhibitor U0126 was evaluated using a Boyden chamber. *P < 0.01, one-way ANOVA. Data are represented as mean ± SD, *n* = 4 fields.

ROS induce the expression of several genes and activate cell motility in many kinds of cells [7, 44]. As shown in Supplementary Figures 2D and 3A, ROS induced the expression of cell motility-related genes. Cell motilities in both NECs and TECs were activated by generation of ROS and attenuated by ROS inhibition (Figure 3A). We speculated that BGN in TECs was involved in this phenomenon. Since TLR2 and TLR4 were reported as BGN receptors [12, 25, 26, 29], we analyzed cell motility in the presence of TLR2 and TLR4 inhibitors (Figure 3B). As expected, pyocyanin-induced TEC motility was inhibited by TLR inhibitors. On the other hand, NEC motility induced by pyocyanin was not inhibited by TLR inhibition. In contrast, inhibition of VEGF signaling reduced cell motility in both NECs and TECs (Supplementary Figure 3B). These data suggest that activated TEC motility by ROS accumulation was at least partially mediated by BGN and TLR, whereas activation of NEC motility by ROS was not.

To examine the contribution of BGN to cell motility in TECs, *Biglycan* expression in TECs was knocked down by shRNA (Figure 3C). In TECs with BGN knockdown, the number of migrated TECs was not increased in response to pyocyanin treatment (Figure 3D). NF-κB and ERK1/2 are downstream targets of the BGN-TLR2/4 signaling pathway [25, 26, 29]. To clarify the potential intracellular signaling cascade by which BGN stimulates TEC migration, we next examined NF-κB and ERK1/2. Phosphorylation of NF-κB was attenuated by BGN knockdown in TECs (Figure 3E). TEC motility was also inhibited by the NF-κB inhibitor BAY11-7082 (Figure 3F). Similarly, the activation of ERK1/2 was abolished by BGN knockdown in TECs (Figure 3G). Furthermore, the number of migrated TECs decreased upon treatment with U0126, a specific inhibitor of mitogen-activated protein kinase (MEK) 1 and 2 (Figure 3H). We previously revealed that exogenous BGN treatment rescued the migration of TECs in which endogenous BGN was knocked down [12]. In addition, when BGN was treated in BGN knockdown TECs, both NF-κB and ERK were activated (Supplementary Figure 3C and 3D). Taken together, these data suggest that the NF-κB and ERK pathways are involved in activated TEC motility with upregulation of BGN.

### Anti-angiogenic effect by ROS inhibition via downregulation of BGN

**Figure 4:**
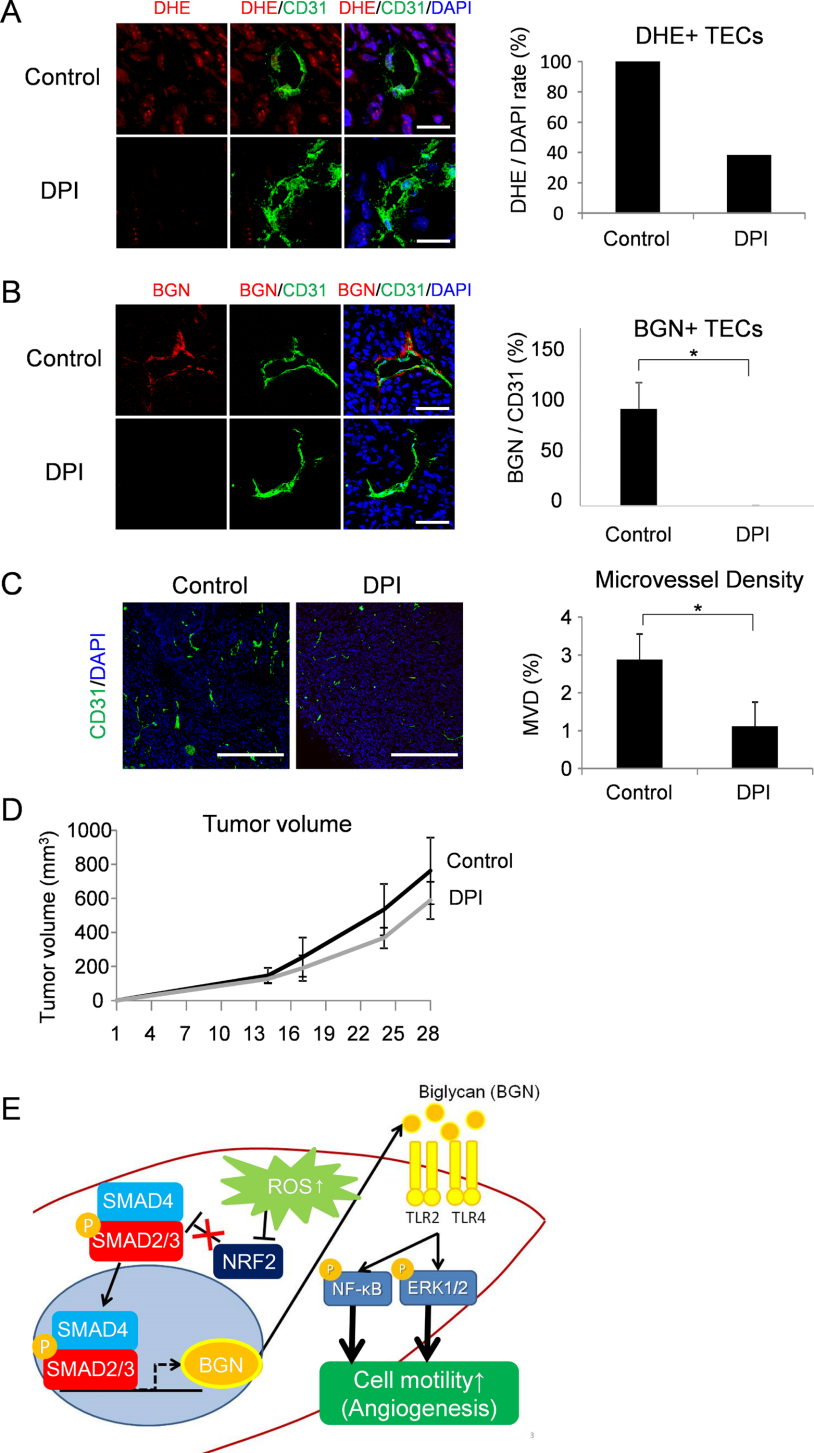
Anti-angiogenic effect by ROS inhibition via downregulation of BGN (**A**) ROS were visualized with DHE staining (red) in tumor tissues of DPI-treated mice. Merged images stained with anti-CD31 antibody (green) and DAPI (blue) are also shown. DHE-stained CD31 positive blood vessels were counted. Scale bar = 30 μm. (**B**) Representative tumor tissues were fixed, sectioned and stained with the anti-CD31 antibody (green) and the anti-BGN antibody (red). BGN^−^positive blood vessel density in each group was analyzed by Image J. Scale bar = 60 μm. *P < 0.01, two-sided Student's *t*-test. Data are mean ± SD, *n* = 5 fields. (**C**) Tumor cryosections were stained with anti-CD31 antibody (green) and counterstained with DAPI (blue). CD31 positive blood vessel density was analyzed in both groups by Image J. Scale bar = 500 μm. *P < 0.01, two-sided Student's *t*-test. Data are mean ± SD, *n* = 10 fields. (**D**) Images of resected tumors (upper panel). A375-SM tumor cells were inoculated subcutaneously into two groups of nude mice; each day, controls (*n* = 4) were injected with DMSO and the test group (*n* = 4) was injected with DPI (2 mg/kg). Tumor volume was measured in the indicated days (*P* = 0.157, two-sided Student's *t*-test.) (**E**) Schematic model. Elevated ROS in TECs downregulates NRF2 expression, which results in activation of SMAD2/3. Activated SMAD2/3 induces BGN expression, which signals TEC migration through TLR2 and TLR4 in an autocrine manner via NF-κB and ERK1/2 activation.

Since BGN contributes to angiogenesis in TECs [12] and BGN was upregulated by ROS accumulation as described above, we treated tumor-bearing mice with DPI, a ROS inhibitor, to examine potential anti-angiogenic effects. ROS inhibition by DPI in tumor tissues was confirmed by DHE staining (Figure 4A). ROS in tumor blood vessels also reduced by DPI treatment (Figure 4A). Consistent with the *in vitro* data, BGN levels in TECs were reduced by ROS inhibition (Figure 4B). Furthermore, the microvessel density, as determined by CD31 staining, was decreased by inhibition of ROS (Figure 4C). Tumor size did not change significantly in response to DPI treatment, but there was a tendency of an anti-tumor effect (Figure 4D). These data suggest that accumulation of ROS in tumors accelerates tumor progression with activation of tumor angiogenesis via BGN (Figure 4E).

## DISCUSSION

In this study, we found that BGN, a marker for TECs, is upregulated by ROS induction of NRF2 downregulation and subsequent increase of phosphorylation of SMAD2/3. High expression of BGN induces high motility in TECs via NF-κB and ERK1/2 activation in an autocrine manner. ROS inhibition induces antiangiogenic effects with downregulation of BGN in tumor blood vessels *in vivo*.

Whether the inhibition (or activation) of ROS causes antitumor effects has remained controversial. Generation of ROS causes DNA damage, which results in cell death [6]. On the other hand, low concentrations of ROS are required for signal transduction pathways to mediate cell growth, migration and differentiation, even in normal cells [6, 7]. Many studies have demonstrated accumulation of ROS in tumor tissues. Cancer cells develop ROS resistance with accelerated metabolism [6, 8]. ROS is also involved in tumor progression and neovascularization with activating redox signaling pathways [6, 7]. Only a few reports have focused on the effects of ROS on tumor blood vessels. Okuno *et al* reported that ROS accumulated in tumor blood vessels with activation of ATM [23]. The authors detected high levels of ROS in freshly isolated TECs of B16 tumors compared with normal counterparts. Similarly, our data showed *in vivo* ROS accumulation in tumor blood vessels analyzed by DHE staining. However, our results showed that ROS levels were almost the same in cultured NECs and TECs. We assumed that ROS may be generated in *in vivo* endothelial cells caused by their surrounding microenvironment as quick response. However, unlike tumor cells, TECs do not exhibit constitutively increased ROS levels. The high levels of ROS in tumor cells are caused by characteristic changes in several pathways that influence ROS accumulation [8]. Alterations in these pathways are not observed in TECs.

NRF2 is a master regulator of the antioxidant defense pathway [8, 35] and is activated in cells upon exposure to oxidative stress. Mutations in NRF2 are found in some cancers, and these mutations enhance NRF2 activity, which causes resistance to oxidative stress [35]. We have not analyzed the mutation status of NRF2 in TECs in this study, but our results showed that NRF2 was elevated in TECs compared with NECs, even in the basal cultured conditions. NRF2 expression in NECs was increased by the ROS inducer. However, contrary to previous reports that showed increased NRF2 in response to ROS, our results showed that NRF2 levels were decreased by ROS in TECs. Although further analyses are required to elucidate the mechanism for NRF2 upregulation in TECs under basal conditions, this novel finding encouraged us to investigate the effect of NRF2 expression on pro-angiogenic properties in TECs, which is a distinct characteristic of these cells.

We previously reported that TECs exhibit a pro-angiogenic phenotype with upregulation of BGN [12]. Since SMAD2/3 are regulators of BGN [41] and NRF2 negatively regulates the phosphorylation of SMAD2/3 [36, 37], we examined the regulation of BGN expression by ROS accumulation via NRF2. As expected, BGN expression in TECs correlated with activation of SMAD2/3. However, the level of both ROS and phosphorylation of SMAD2/3 were almost the same in NECs and TECs under untreated conditions, although BGN expression was different. These results suggest that BGN expression in untreated conditions was not due to activation of SMAD2/3. Indeed, we found that the *Biglycan* promoter is demethylated in TECs [29]. This indicates that epigenetic regulation may also contribute to regulation of BGN expression in addition to ROS.

We previously reported that ROS caused abnormality in endothelial cells [22]. Our results also showed that treatment of tumor-bearing mice with a ROS inhibitor reduced the number of tumor blood vessels with downregulation of BGN expression. Together these findings suggest that control of ROS levels could be a target for new anti-angiogenic and anticancer therapy.

## MATERIALS AND METHODS

### Ethics

Investigation has been conducted in accordance with the ethical standards and according to the Declaration of Helsinki and according to national and international guidelines and has been approved by the Institutional Ethics Committee of Hokkaido University.

### Human tissue samples

Tumor tissues and corresponding normal tissues were surgically resected from patients who were clinically diagnosed with hepatocellular carcinoma, colorectal cancer or renal cell carcinoma at Hokkaido University Hospital. All protocols were approved by the Institutional Ethics Committee of Hokkaido University and written informed consent was obtained from each patient before surgery. Final pathological diagnosis of the cases was confirmed by examination of formalin-fixed surgical specimens.

### Cell culture

Human malignant melanoma A375-SM (super metastatic) cells were kindly provided by Dr. Fidler (M.D. Anderson Cancer Centre, Houston, TX, USA). A375-SM cells were authenticated by JCRB Cell Bank (Ibaraki, Japan) on January 2014 and cultured in Minimum Essential Medium (MEM, GIBCO, Thermo Fisher Scientific, Waltham, MA, USA) supplemented with 10% heat-inactivated fetal bovine serum (FBS). TECs and NECs were isolated as previously described [9, 11, 12, 14, 16–21, 40]. Briefly, A375-SM tumor cells were subcutaneously injected into 6-week-old female nude mice (BALB/c AJcl-nu/nu, CLEA Japan, Tokyo, Japan), which were housed under specific pathogen-free conditions. All procedures for animal care and experimentation adhered to institutional guidelines and were approved by the local animal research authorities. A375-SM tumors grown in nude mice and the dermis of non-tumor bearing mice were minced, and endothelial cells were sorted using a magnetic cell sorting system with CD31 microbeads (Miltenyi Biotec, Bergisch Gladbach, Germany). Endothelial cells (ECs) were then maintained in EGM-2 MV (Lonza, Basel, Swiss) containing 15% FBS. To eliminate any remaining human tumor cells that expressed heparin-binding EGF-like growth factor, a diphtheria toxin (DT) receptor [9, 11, 12, 14, 16–21, 40], 500 ng/mL of DT (Merck Millipore, Darmstadt, Germany) was added to EC subcultures. Isolated ECs were further purified using FITC-BS1-B4-lectin, and used for experiments after the expression of EC markers and tube formation ability as EC phenotypes were confirmed [9, 11, 12, 14, 16–21, 40]. Cells were cultured at 37°C in a humidified atmosphere containing 5% CO_2_.

### Dihydroethidium (DHE) staining and immunostaining

For immunohistochemistry, frozen sections were incubated with 10 μM of DHE (Sigma-Aldrich, St, Louis, MO, USA) for 30 min at room temperature. After blocking with 5% goat serum in PBS, Alexa Fluor® 647 anti-CD31 antibody (Biolegend, San Diego, CA, USA) was added to visualize blood vessels. For immunocytochemistry, cells were seeded on cover glasses in 5% FBS containing EBM2 (Lonza) and incubated for 24 h to allow for cell adhesion. After incubation with 25 μM of pyocyanin (SIGMA) to induce ROS, cells were fixed with 4% paraformaldehyde. After blocking with 5% goat serum in PBS, SMAD2/3 (D7G7) XP^®^ Rabbit mAb (Cell Signaling Technology, Beverly, MA, USA) or anti-NRF2 antibody (Abcam Inc., Cambridge, MA, USA) were added followed by incubation with Fluor^®^ 647-goat anti-rabbit IgG antibody (Invitrogen Life Technologies). F-actin was stained with MFP488 Phalloidin (MoBiTec GmbH, Germany). Counterstaining was performed using 4,6-diamidino-2-phenylindole (DAPI; Roche, Diagnostics, Mannheim, Germany). Sample images were acquired using a FV1000 confocal microscope (Olympus, Tokyo, Japan). The acquired images were processed using Fluoview FV10-ASM Viewer software (Olympus). Microvessel density (MVD) of each CD31-stained tumor was determined as previously described [14, 16, 19]. For quantitative analysis, the number of SMAD2/3 positive cells stained in DAPI areas was counted as SMAD2/3 nuclear localization. Image J software from the NIH (Bethesda, MD, USA) was used to quantify the NRF2 stained areas; the quantification was normalized with F-actin stained area.

### Measurement of ROS levels

After incubation with EBM-2 containing 5% FBS for 16 h, cells (3 × 10^3^ cells/well) were seeded into 96-well dishes in EBM-2 containing 5% FBS with 25 μM of pyocyanin for 30 min. The Total ROS/Superoxide detection kit (Enzo Life Science, Plymouth Meeting, PA) and Varioskan Flash (Thermo Fisher Scientific) were used to measure ROS levels. After incubation of ECs with EBM-2 supplemented with 5% FBS (control) or 0.5% FBS (serum starvation) for 24 h, the Total ROS/Superoxide detection kit (Enzo Life Science) and FACS Aria II (BD Biosciences, San Jose, CA, USA) were used to measure the ROS levels. After incubation of ECs in normoxia (20% O_2_) or hypoxia (1% O_2_) for 24 h, ROS levels were measured with the Total ROS/Superoxide detection kit.

### Cell migration assay

EC motility was analyzed with a Boyden chamber as previously described, with some modifications [12, 16]. After incubation of ECs with 25 μM of pyocyanin for 24 h, ECs were treated with TLR2 antibody, TLR4 antibody, VEGF antibody or Ki8751 for 90 min prior to assays. Cells were placed in the upper chamber and 5% FBS EBM2 was placed in lower chamber. After 6 h incubation, non-migrating cells were removed with a cotton swab, followed by fixation with 10% formalin (Wako, Tokyo, Japan) and staining with Mayer's hematoxylin (Wako). Cells that had migrated to the bottom surface were counted using a microscope.

### Quantitative real-time RT-PCR

Total RNA was extracted using the ReliaPrep™ RNA Cell Miniprep System (Promega Corporation, Madison, WI, USA) according to the manufacturer's instructions. cDNA was synthesized using ReverTra-Plus (Toyobo, Osaka, Japan), Oligo dT primer, Random hexamer (Hokkaido System Science Co. Ltd., Sapporo, Japan) and dNTP mixture (Takara Bio, Kusatsu, Japan) as previously described [16] and amplified by PCR. Quantitative real-time RT-PCR was performed using the KAPA SYBR® FAST qPCR Kit (KAPA Biosystems, Boston, MA, USA) according to the manufacturer's instructions. Cycling conditions were set based on CFX Manager (Bio-Rad, Hercules, CA, USA). mRNA expression levels were normalized to that of *Actb*. The primers used are as follows: *Actb*: FW: 5′-TTTGCACATGCCGGAGCCGTTG-3′, RV: 5′-TTTGCAGCTCCTTCGTTGCCGG-3′, *Biglycan*: FW: 5′-AACTCACTGCCCCACCACAGCTTC-3′, RV: 5′-GC GGTGGCAGTGTGCTCTATCCATC-3′, *Pai1*: FW: 5′-C CACAAAGGTCTCATGGACCAT-3′, RV: 5′-TGAAA GTGTTGTGCCCTCCAC-3′, *Smad7*: FW: 5′-GAAAAG GAAAGGAAGACCGGCTG-3′, RV: 5′-CTCGTGAG ATGTCTGGAGGGTC-3′, *Nrf2*: FW: 5′-TCTTGCCCT AGCCTTTTCTCCG-3′, RV: 5′-AACTAGGAGATAGC CTGCTCGC-3′, *Ho-1*: FW: 5′-CAGCCCCACCAAG TTCAAACAG-3′, RV: 5′-CTCAATGTTGAGCAGGAA GGCG-3′. *Smad2*: FW: 5′-CGCCTTGGTTGTCAGTT GATCC-3′, RV: 5′-ACTCTCCAGGAAAGAGGACACC -3′, *Vegf-a*: FW: 5′- CCTGCCGAAGCTCTCCACGAT TT-3′, RV: 5′- AGAACACTTGTTGCAGGCAGCGG-3′, *Vegfr2*: FW: 5′- GCCCTGCTGTGGTCTCACTAC-3′, RV: 5′- CAAAGCATTGCCCATTCGAT-3′, *Cxcr7*: FW: 5′- CTACAAACTGCTCAGCACTGAAGG-3′, RV: 5′- TGCCAGTCAATTCCCAGTTGCCCG -3′, *Ptgir*: FW: 5′- AGGCAGAGGTGCTGGAGGGTCTAGA-3′, and RV: 5′- TCGCAGGAGACAACCTGGTC-3′.

### Western blotting

Cells were lysed using RIPA buffer (Cell Signaling Technology). Total protein concentration was determined using a BCA Protein Assay kit (Pierce, Rockford, IL, USA). Western blotting was performed according to standard methods using antibodies specific for NRF2 (Abcam Inc., Cambridge, MA, USA), pSMAD2/3, SMAD2/3, pNF-κB, NF-κB, pERK, ERK, β-Actin (Cell Signaling Technology) and an HRP-conjugated secondary antibody as previously described [15, 16, 29]. Quantitative analysis was performed using Image J.

### RNA interference

*Nrf2* siRNA (*Nrf2* si#1: 5′-UGUUUGACUUUAGUC AGCGACAGAA-3′, 5′-UUCUGUCGCUGACUAAAG UCAAACA-3′, *Nrf2* si#2: 5′-GCAUGUUACGUGAU GAGGAUGGAAA-3′, 5′-UUUCCAUCCUCAUCACGU AACAUGC-3′) and *Smad2* siRNA (*Smad2* si#1: 5′-CAGGACGGUUAGAUGAGCUUGAGAA-3′, 5′-UU CUCAAGCUCAUCUAACCGUCCUG-3′, *Smad2* si#2: 5′-UCGGAACCUGCAUUCUGGUGUUCAA-3′, 5′-UU GAACACCAGAAUGCAGGUUCCGA-3′) were intro- duced into cells using Lipofectamine^®^ RNAiMAX Reagent (Thermo Fisher Scientific). Non-targeting Stealth^TM^ RNAi Negative Control Duplexes (Thermo Fisher Scientific) was used as a negative control. BGN knockdown was performed as previously described [29]. Briefly, the following oligonucleotides were annealed, digested with BglII and XbaI and inserted into the BglII-Xbal sites of the Gateway entry vector pENTR4-H1 (Invitrogen): 5′-GATCTCCgaacatagcc agatgaagaTTCAAGAGAtcttcatctggctatgttcTTTTTGGAA T-3′, 5′-CTAGATTCCAAAAAgaacatagccagatgaagaTC TCTTGAAtcttcatctggctatgttcGGA-3′ for *shBiglycan*#1; 5′-GATCTCCgttcactacctgtcaatccTTCAAGAGAggattgaca ggtagtgaacTTTTTGGAAT”-3′, and 5′-CTAGATTC CAAAAAgttcactacctgtcaatccTCTCTTGAAggattgacagg tagtgaacGGA-3′ for *shBiglycan*#2. Sequences against GFP were used as a negative control. These entry clones were then transferred in the lentiviral vector (from H. Miyoshi) and packaging vector pCAG-HIVgp and the VSV-G- and REV-expressing construct pCMV-VSV-G-RSV-REV (from H. Miyoshi) were introduced into 293T cells using FuGeneHD (Promega), according to the manufacturer's recommendations. Lentivirus-mediated gene transfer was performed, as previously described [29].

### *In vivo* tumor model

1 × 10^6^ cells of A375-SM tumor cells were subcutaneously injected into the right flanks of 7-week-old female nude mice (BALB/c AJcl-nu/nu) obtained from CLEA Japan. Each mouse received a single daily intraperitoneal injection of DPI (2 mg/kg), with dimethyl sulfoxide (DMSO) diluted by PBS (vehicle) injected as control. Animals were monitored for 28 days.

### Statistics

Data expressed as the mean ± standard deviation (SD) were performed in triplicate and subjected to one-way ANOVA, followed by a Tukey–Kramer multiple comparison test. A two-sided Student *t*-test was used for comparison between two groups.

## SUPPLEMENTARY MATERIALS AND FIGURES


