# Supplementary material for: ROS enhance angiogenic properties via regulation of NRF2 in tumor endothelial cells
Source: Oncotarget. 2017 May 2;8(28):45484–95. doi: 10.18632/oncotarget.17567 (PMC5542202; doi:10.18632/oncotarget.17567)
Supplement: Supplementary file 1 [file oncotarget-08-45484-s001.pdf]

ROS enhance angiogenic properties via regulation of NRF2 in tumor endothelial cells

Supplementary Materials

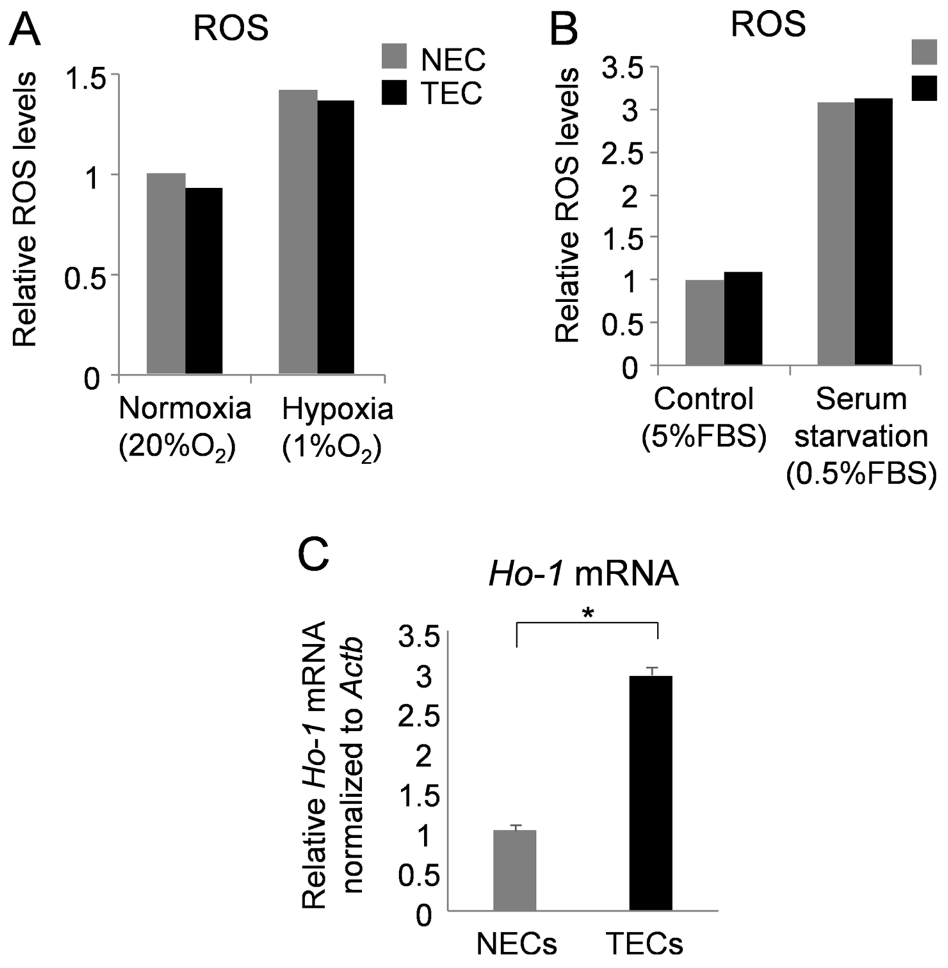

Supplementary Figure 1: (**A**) Relative ROS levels in NECs (gray columns) and TECs (black columns) cultured in normoxia (20% O_2) or hypoxia (1% O_2) condition for 24 h. (**B**) Relative ROS levels in NECs (gray columns) and TECs (black columns) in control (medium with 5% FBS) or starved condition (medium with 0.5% FBS) for 24 h. (**C**) *Ho-1* mRNA in NECs and TECs were analyzed by real-time PCR. *$P < 0.01$, two-sided Student's *t*-test. Data are mean ± SD, $n = 4$ real-time RT-PCR runs.

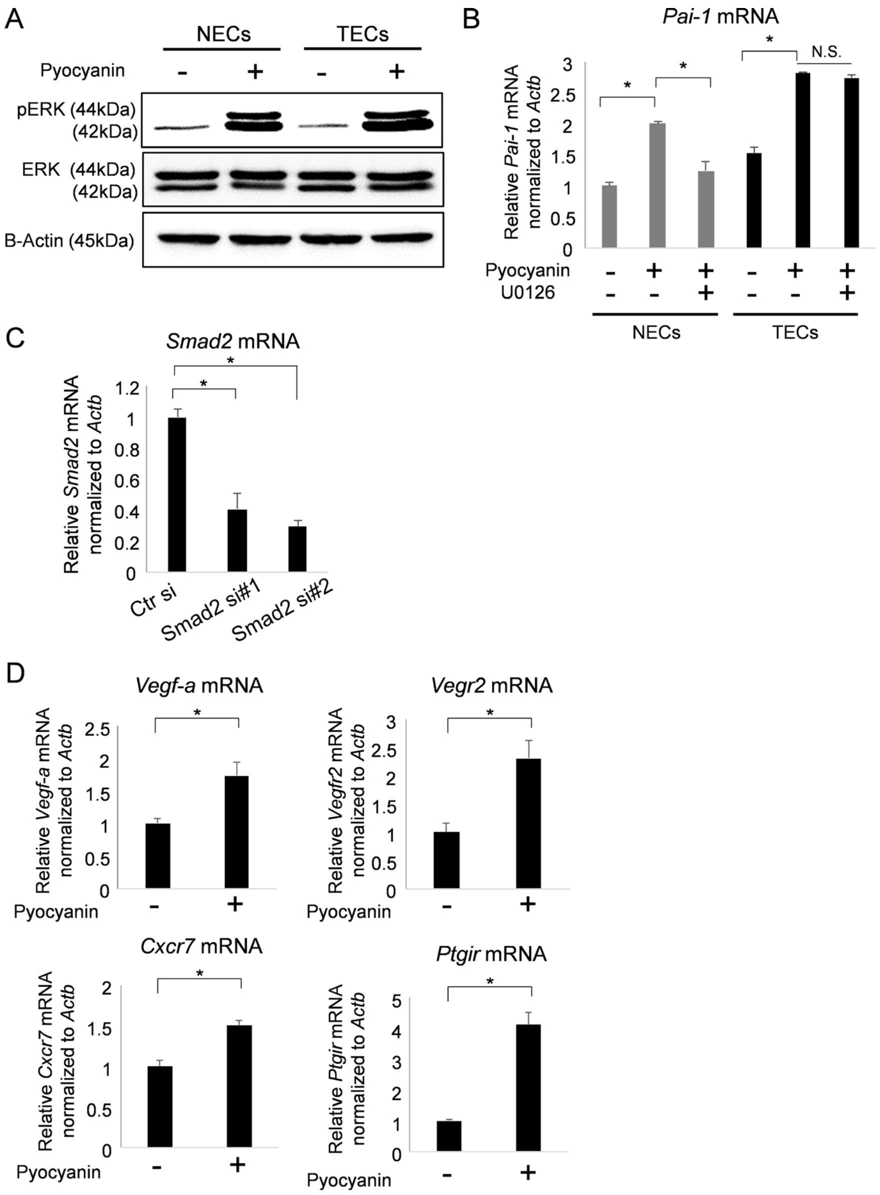

Supplementary Figure 2: (**A**) Phosphorylated ERK1/2 (pERK) and total ERK1/2 (ERK) levels were analyzed in ECs stimulated with or without pyocyanin (25 μM) by western blotting. B-Actin served as loading controls. (**B**) *Pai-1* mRNA in NECs and TECs with or without pyocyanin (25 μM) or the MEK inhibitor U0126 (1 μM) were analyzed by real-time PCR. *$P < 0.01$, N.S., not significant. One-way ANOVA. Data are mean ± SD, $n = 4$ real-time RT-PCR runs. (**C**) Real-time PCR confirms silenced *Smad2* mRNA levels. *$P < 0.01$, one-way ANOVA. Data are mean ± SD, $n = 4$ real-time RT-PCR runs. (**D**) *Vegf-a*, *Vegfr2*, *Cxcr7* and *Ptgir* mRNA expressions in TECs with or without pyocyanin treatment (25 μM) were analyzed by real-time PCR. *$P < 0.01$, two-sided Student's *t*-test. Data are mean ± SD, $n = 4$ real-time RT-PCR runs.

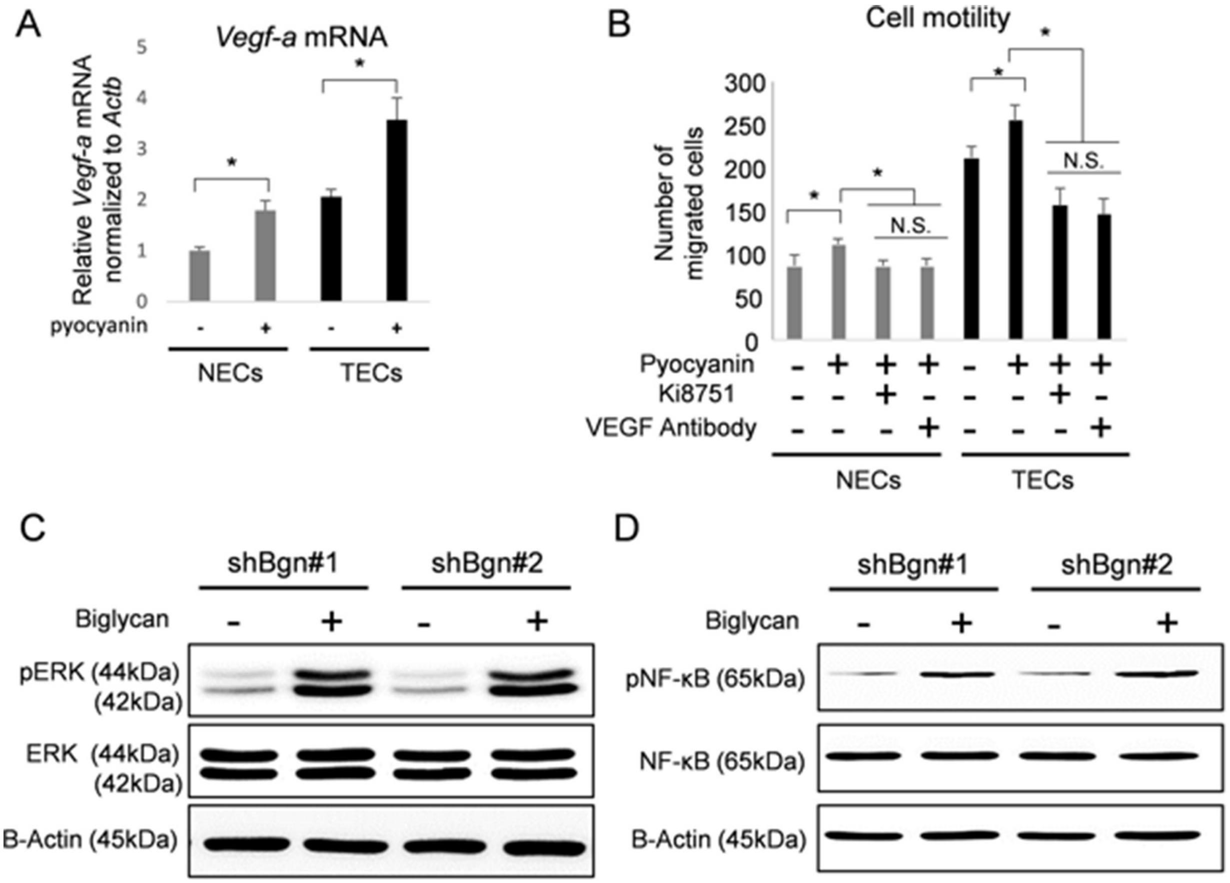

Supplementary Figure 3: (**A**) *Vegf-a* mRNA in NECs and TECs with or without pyocyanin treatment (25 μM) were analyzed by real-time PCR. *$P < 0.01$. One-way ANOVA. Data are mean ± SD, $n = 4$ real-time RT-PCR runs. (**B**) Cell motilities of NECs and TECs treated with pyocyanin (25 μM) were analyzed by Boyden chamber migration assay in the presence of anti-VEGFA antibody (150ng/ml) or VEGFR2 kinase inhibitor, Ki8751 (10 nM). *$P < 0.01$, one-way ANOVA. Data are represented as mean ± SD, $n = 4$ fields. (**C**) Phosphorylated ERK (pERK) and total ERK (ERK) levels were analyzed in *Biglycan* knockdown TECs stimulated with BGN (1 μg/ml) by western blotting. B-Actin served as loading controls. (**D**) Phosphorylated NF-κB (pNF-κB) and total NF-κB (NF-κB) in *Biglycan* knockdown TECs stimulated with BGN (1 μg/ml) by western blotting. B-Actin served as loading controls.
